# Self-Assembly of Silver Nanowire Films for Surface-Enhanced Raman Scattering Applications

**DOI:** 10.3390/nano13081358

**Published:** 2023-04-13

**Authors:** Yanzhao Pang, Mingliang Jin

**Affiliations:** 1 South China Academy of Advanced Optoelectronics, South China Normal University, Guangzhou 510006, China; 2 International Academy of Optoelectronics at Zhaoqing, South China Normal University, Zhaoqing 526060, China

**Keywords:** silver nanowire, self-assembly, surface enhanced Raman scattering applications

## Abstract

The development of SERS detection technology is challenged by the difficulty in obtaining SERS active substrates that are easily prepared, highly sensitive, and reliable. Many high-quality hotspot structures exist in aligned Ag nanowires (NWs) arrays. This study used a simple self-assembly method with a liquid surface to prepare a highly aligned AgNW array film to form a sensitive and reliable SERS substrate. To estimate the signal reproducibility of the AgNW substrate, the RSD of SERS intensity of 1.0 × 10^−10^ M Rhodamine 6G (R6G) in an aqueous solution at 1364 cm^−1^ was calculated to be as low as 4.7%. The detection ability of the AgNW substrate was close to the single molecule level, and even the R6G signal of 1.0 × 10^−16^ M R6G could be detected with a resonance enhancement factor (EF) as high as 6.12 × 10^11^ under 532 nm laser excitation. The EF without the resonance effect was 2.35 × 10^6^ using 633 nm laser excitation. FDTD simulations have confirmed that the uniform distribution of hot spots inside the aligned AgNW substrate amplifies the SERS signal.

## 1. Introduction

Surface-enhanced Raman spectroscopy (SERS) can identify analytes by their unique molecular vibrational signals, serving as a powerful analytical technique [1]. SERS enables a fast, sensitive, label-free, multiplex, and nondestructive analysis with tremendous amplified Raman scattering by analytes in “hot spot” areas [2,3]. SERS spectroscopy, therefore, develops into an effective analytical technique used in bio-medicine [4], analytical science [5], and material physics [6], which attracts increasing research enthusiasm [7]. During exposure to laser spots, probe molecules close enough to a nano-structured coinage metal surface can bring strong enhanced Raman scattering signals. This phenomenon has been explained by the electromagnetic (EM) enhancement theory centered on the localized surface plasmon resonance (LSPR) effect and the chemical enhancement (CE) theory represented by the charge transfer mechanism [8]. Furthermore, it has been shown that the EM mechanism plays a decisive role in SERS [9]. EM theory identifies three types of “hot spot” structures with 1–10 nm feature sizes: nanogaps, nanocrevices, and nanotips [10]. The quantity and quality of the “hot spot” on the substrate determine the SERS performance of the substrate and further SERS detection capabilities. Thus, how to prepare high-performance substrates with an ample “hot spot” has become an important issue.

There are typically two primary categories of substrates: colloidal nanoparticles and chip-based substrates. Although the electromagnetic (EM) field enhancement factors (EF) of the former kind can reach as high as 10^12^, they are unstable and cannot firmly capture target molecules in the “hot spot” [11]. In contrast, signal reproducibility and reliability deserve attention for achieving quantitative data, especially in practical applications [12,13,14,15]. The chip-based periodic nanostructure offers the possibility to gain reproducible and reliable Raman signals. Although nano-fabrication methods, such as electron-beam lithography (EBL) [16,17,18] and nanoimprinting lithography (NIL) [19,20,21], can be applied to fabricate substrates with a well-ordered nanostructure, they either require expensive equipment or prefabricated high-density and precise templates. The previous research results have shown that preparing a desired SERS substrate with an ordered “hot spot” that satisfies all requirements of high stability, high sensitivity, low cost, and convenience is still challenging. Meanwhile, interfacial self-assembly is a versatile and efficient method for forming periodic nanoarrays from plasmonic nanoparticles of varying sizes, shapes, and compositions [22,23,24]. Nanosphere lithography (NSL) [25,26,27], relying on self-assembly methodologies, has emerged as a promising approach for fabricating meticulously organized nanoarrays. Nonetheless, its implementation often mandates transforming untainted organic or inorganic materials into SERS-active materials, engendering intricacies in the procedural workflow and undermining the accuracy of structural control. Among those nanoparticles, Ag nanowire (AgNW)-based substrates [28,29] with high aspect ratios have unique advantages, including outstanding free electron supply capabilities, good stability, and expandable flexibility. Furthermore, previous research has shown that aligned AgNWs can effectively adsorb probe molecules for violently enhanced detection signals with a uniform surface [30]. Researchers have achieved the preparation of aligned silver AgNW thin films. Considering that the microfluidic approach [31,32] and electro-hydrodynamic jet printing [33] are sophisticated and not conducive to large-scale applications, methods such as working with the Langmuir–Blodgett (LB) technique [34,35] on a simple device are preferred.

Within this study, we reported the oil-water-air three-phase interface to fit the self-assembly of long AgNWs and finally create an ultrasensitive molecular detection SERS substrate with a high-density “hot spot” due to the nanogaps between neighboring AgNWs. Rhodamine 6G (R6G) was applied as the probe molecule to determine the detection capability of the prepared substrate. It has shown an ultra-high detection sensitivity with a detection limit of 10^−16^ M and a good signal reproducibility excited by the resonance laser wavelength. Even without the resonance excitation environment, the EF is still 2.35 × 10^6^ (Supplementary Materials, Figure S3).

## 2. Materials and Methods

### 2.1. Self-Assembly of AgNWs Films

**Figure 1:**
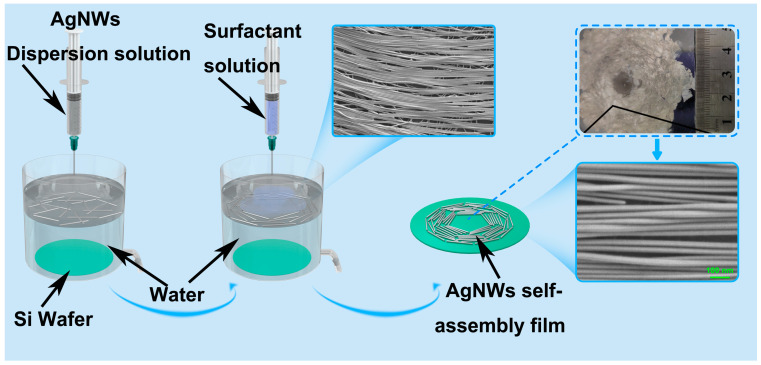
Schematic of the process for fabricating aligned-AgNWs Si substrate.

AgNW-based films were prepared with commercial nanowires from Zhejiang Kechuang Advanced Materials Technology Co., Ltd in Hangzhou, China, (diameter 45 nm ± 10 nm, length 10–100 μm) (Supplementary Materials, Figure S1). They are synthesized using a polyvinylpyrrolidone (PVP)-mediated method. After ultrasonic and centrifugal cleaning of the nanowires with pure water, we dispersed the silver nanowires in a solution by adding a certain mass of ethanol and n-Hexane. Then, the dispersion, which contains AgNWs, was injected at a speed of 0.25 mL/min using a syringe onto a round water surface with a diameter of 100 mm. The self-assembly AgNWs were completed during the evaporation of the non-polar solution. After the water was drained, the Ag NWs films were deposited on hydrophilic silicon wafers freshly treated with plasma treatment. The schematic diagram is shown in Figure 1.

### 2.2. Morphology Characterization

The morphology of the fabricated AgNW films was analyzed by high-resolution scanning electron microscopy (HR-SEM, GeminiSEM 500, Carl Zeiss Microscopy GmbH). The atomic force microscopy (AFM) test was performed using Bruker AFM Dimension ICON with OPUS 240 AC-NA probes in tapping mode. The experimental environment of choice is air to ensure an accurate data acquisition, and various scan parameters were set. The scan size was established at 297 nm, and the scan angle was set to 0. The amplitude setpoint was adjusted to optimize the setpoint. Until the trace and retrace scan lines displayed minimal variation, the after-integral gain and proportional gain were optimized. Finally, the scan rate was established at 0.996 Hz to ensure the reliability of the acquired data.

### 2.3. Raman Spectroscopy Measurement

To prepare the R6G stock solution, 48 mg of R6G was weighed and added to 100 mL of water, resulting in a 10^−^^3^ M stock solution. Then, using a pipette, 1 mL of the stock solution was added to 9 mL of deionized (DI) water to obtain a 10^−^^4^ M R6G solution. This process was repeated to form R6G solutions of different concentrations ranging from 10^−^^7^ to 10^−^^16^ M.

The SERS substrate was immersed in a 3-cm-diameter glass petri dish containing 5 mL of probe molecules for one hour, washed three times in DI water, and then blown dry for SERS detection. Raman scattering measurements were acquired using a Finder Insight Raman instrument. A pulsed laser source with 532 nm radiation was applied in the Raman instrument with a 50× objective (NA = 0.55). The diameter of the laser spot is 10 μm. The excitation light power was set to 0.4 mW for measuring R6G samples. The integration time was set to 0.05 s.

The Raman shift was calibrated using single crystal silicon before the test, and the power was set to 5%, approximately equal to 1 mW, with an integration time of 0.3 s. To detect R6G on AgNWs substrates, we set the power to 2%, corresponding to 0.4 mW, and the integration time to 0.05 s. Raman spectra were then recorded by focusing on the SERS substrate using the Finder Insight Raman instrument with the software named INScan-M and output in txt type. Then, the data is copied and imported into the Origin software to plot the spectra without extra processing. 

Two samples were tested for each concentration of the probe molecule, with 20 points per sample, including 30 points for 10^−^^10^ M R6G, by randomly moving the stage of the Raman instrument. 

### 2.4. Finite Difference Time Domain (FDTD) Simulations

The FDTD simulation was conducted using Finite Difference IDE software (Anasys lumerical). The nanostructure composed of three AgNWs (length of 500 nm, diameter of 45 nm) was modeled as aligned AgNW films. A perfectly matched layer was applied as a boundary condition in the FDTD option list. The object was illuminated by a total-field scattered field (TFSF) source with a wavelength of 532 nm emitting along the *z*-axis. The mesh dimension was set as 0.3 nm× 0.3 nm × 0.2 nm for the nanogap region in the electric-field monitor. 

## 3. Results and Discussion

### 3.1. The Optimal AgNW Concentration to Obtain Aligned AgNW Self-Assembly Films

The fabrication of AgNW self-assembly films begins by injecting the AgNW dispersion solution into water. When a dispersion droplet touches the water surface, the solution is separated: water-miscible ethanol dissolves into water, whereas a water-immiscible solvent (i.e., n-Hexane) stays on the water (Figure 1). This forms an interface between water (including ethanol) and n-Hexane. AgNWs coated with the side chains of PVP settle at the interface, stabilizing the system by lowering the interfacial energy between water–ethanol and n-Hexane. Dissolving ethanol in water decreases local surface tension, resulting in a circular surface tension gradient near the droplet (Figure 1). This gradient induces a Marangoni flow from the center to the boundary, which drags the floating mass, including n-hexane and AgNWs. The border expands as ethanol spreads out. Thus, the interfacial AgNWs move until they reach the previously transferred mass. Because the Marangoni flow transfers the interfacial AgNWs rapidly, a well-ordered film of AgNWs is formed, and their agglomeration is suppressed. To fully pack the AgNWs on water, 2–3 drops of a surfactant (Triton X-100, 1 wt.%) are added at the center, which pushes the floating AgNWs outward. As a result, AgNWs can be more closely packed. The water-immiscible solvent evaporates within 3 min at room temperature, and a monolayer of assembled AgNW film remains on the water. The AgNW film can be transferred onto various substrates, such as a wafer, a plastic substrate, or an elastomeric substrate, for further processing. An overview of this assembly process is shown in Figure 1.

**Figure 2:**
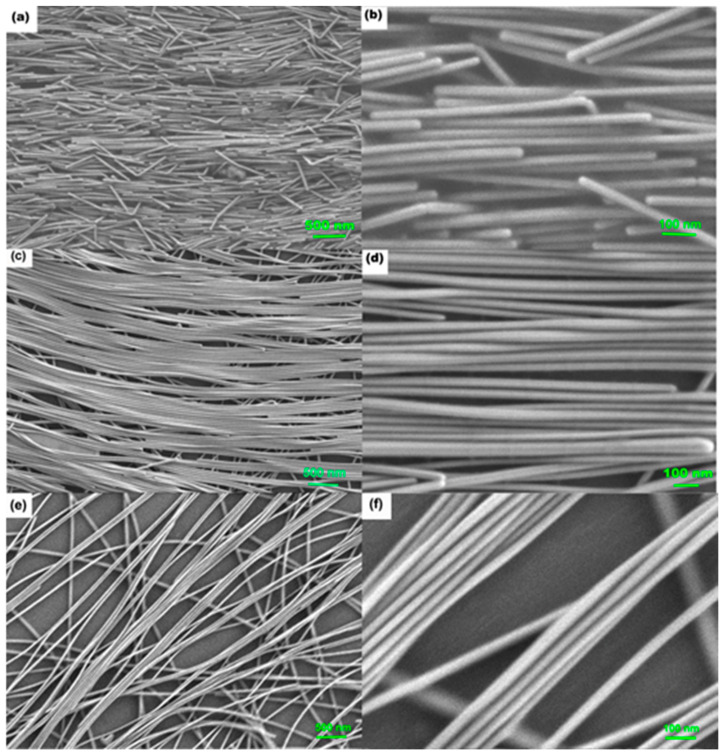
SEM structures of film samples assembled with different concentrations of silver nanowires; (**a**,**c**,**e**) respective large area structure diagram; 2 mg/mL, 1 mg/mL, 0.5 mg/mL. (**b**,**d**,**f**) Respective enlarged detailed structure diagram; 2 mg/mL, 1 mg/mL, 0.5 mg/mL.

Considering that the AgNW film is formed by the Marangoni flow and that the mass transformation plays an essential role in the preparation of aligned AgNW structures, we prepared three different concentrations (weight concentration: 2 mg/mL, 1 mg/mL, 0.5 mg/mL) of AgNW dispersions in ethanol and n-Hexane mixed solution with a volume ratio of 7:3. The morphology of the prepared samples on a 4-inch wafer is shown in Figure 2. Since commercial AgNWs have been coated with the surfactant to avoid agglomeration during storage, after diluting the concentration, we applied ultrasonic washing of the AgNWs with DI water. AgNW suspensions of different concentrations were used to fabricate AgNW films on silicon wafers. From Figure 2a–f, we can see that as the AgNW concentration decreases in the range of 2 mg/mL^−1^ mg/mL, the orderliness of the AgNWs’ arrangement is improved. Additionally, the 1 mg/mL sample tends to form a well-ordered nanowire film compared to the higher-concentration samples. However, the resulting Ag films formed at a lower concentration (0.5 mg/mL) have larger spaces between the arranged AgNWs. The 2-mg/mL-concentration AgNW sample has very short nanowire lengths (about 1 μm), presumably due to the collision between nanowires during ultrasonic cleaning and self-assembly. Meanwhile the substrate prepared using 1 mg/mL AgNW has the most extended length value (>6 μm), which is more conducive to forming aligned nanowire arrays. Furthermore, the 1 mg/mL sample shows fine and uniform nanoslits, which are ideal “hot spots” and distinct features with highly sensitive and reproducible SERS signaling. Based on these results, subsequent Raman measurements are performed using samples prepared at a concentration of 1 mg/mL AgNW. The average diameter of the nanowire is 45 nm ± 10 nm (Supplementary Materials, Figure S4). 

### 3.2. Determining the Reproducibility of Prepared Substrates

**Figure 3:**
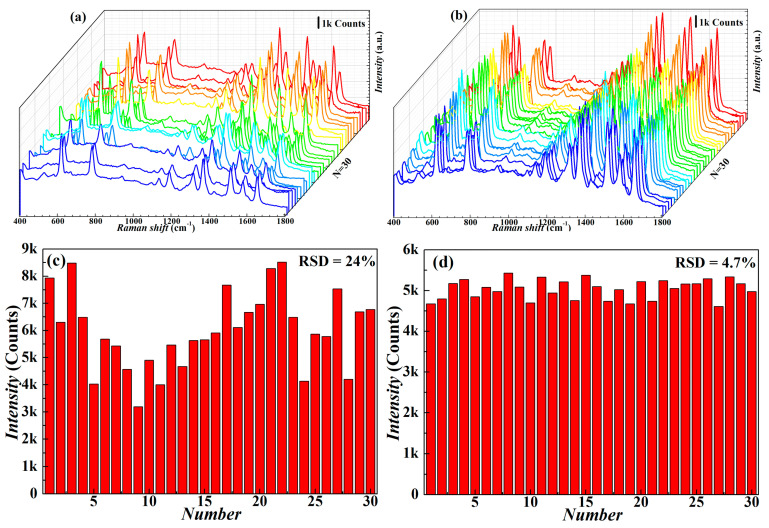
Repeatability and stability of 1.0 × 10^−10^ M R6G on SERS substrates: (**a**,**b**) the Raman spectrum of 30 points randomly selected on AgNWs’ SERS substrate of 2 mg/mL and 1 mg/mL, respectively; (**c**,**d**) Raman intensity distribution at the characteristic peak at 1364 cm^−1^ corresponding to the 30 spectra in (**a**,**b**).

Signal repeatability and stability are essential for evaluating SERS performance, especially for quantitative detection. To study the signal stability and repeatability of the Ag NWs’ SERS substrate, we detected and recorded Raman spectra at 30 random positions on the same substrate using 1.0 × 10^−10^ M R6G as the probe molecule. The data are shown in Figure 3a–d. We counted the peak intensity values to further evaluate the signal repeatability quality and calculated the relative standard deviation (RSD) at the Raman characteristic peak at 1364 cm^−1^ corresponding to the 30 spectra. The more condensed substrate, prepared using 2 mg/mL AgNWs, shows a higher RSD, since there is less surface homogeneity. The better-patterned surface is fabricated, and the lower RSD of the Raman intensity at the Raman characteristic peak at 1364 cm^−1^ is shown to be 4.7%, indicating that the AgNW substrate, manufactured by using the 1 mg/mL AgNWs solution, has excellent signal repeatability and stability.

### 3.3. Hot-Spot Characterization and Enhancement Mechanism of AgNW Substrate

**Figure 4:**
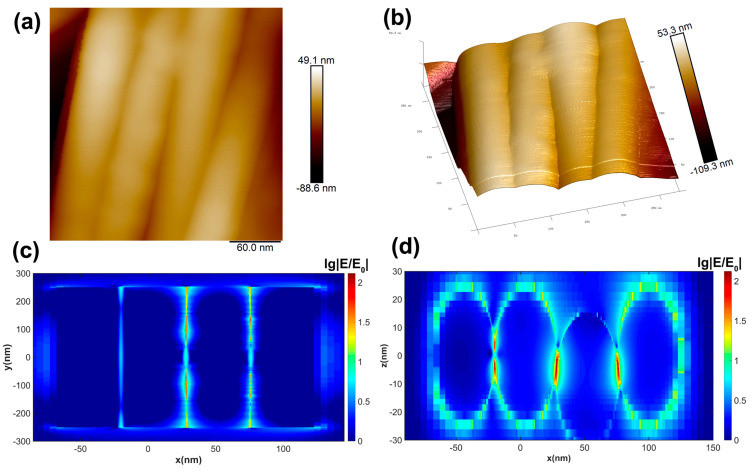
SERS enhancement mechanism of AgNWs substrate: (**a**,**b**) AFM scanned microzone images of AgNW array; (**c**,**d**) normalized logarithmic distribution of electric field intensity based on microzone structure simulated by FDTD, showing the XY cross-section and XZ cross-section.

Based on atomic force microscopy (AFM) scans of microregion structures, it is apparent that AgNW regions are predominantly in contact with each other, forming nanoslits at some locations. The planar and three-dimensional surface morphology of the AFM structure is presented in Figure 4a,b. To investigate the mechanism by which the AgNW substrate enhances Raman spectroscopy through “hot spot” formation, we designed an AgNW structure model similar to the microregion structure. We aligned the nanowires tightly along the X-axis direction, with the third nano tilted at 3° from the horizontal plane, and simulated the system using FDTD software. The electric field intensity distribution in the horizontal and depth cross-sections was recorded utilizing the field monitor and presented in Figure 4c,d. The contact point of the AgNW cross-section contact position created a nanoslit in the horizontal cross-section. Despite the low electric field intensity, a nanoslit with a strong electric field enhancement was formed in the spatial region near the contact point. The depth cross-section revealed that a “hot spot” formed around the forked nanowires creating nanoslits. The presence of nanoslits and nanogaps within a wide range and high density in space provides a theoretical explanation for the high SERS detection sensitivity and signal uniformity enabled by the AgNW substrate for EM enhancement.

### 3.4. Determining Detection Limit and Linear Detection Range 

**Figure 5:**
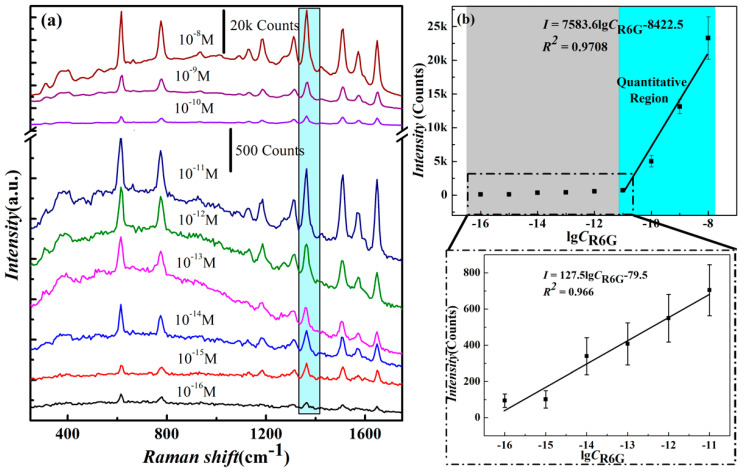
(**a**) SERS spectra of R6G as a function of concentration in the range of 1.0 × 10^−8^ M–1.0 × 10^−16^ M on AgNWs’ SERS substrates; (**b**) SERS intensities at 1364 cm^−1^ for a series of concentrations of R6G ranging from 1.0 × 10^−16^ M to 1.0 × 10^−8^ M. The error bars were obtained with at least five repeated trials.

To detect the sensitivity of the AgNWs’ SERS substrates, R6G was used as the probe molecule, and the concentration range was 1.0 × 10^−8^ M–1.0 × 10^−16^ M. The Raman spectra of 1.0 × 10^−8^ M–1.0×10^−16^ M R6G on the AgNWs’ SERS substrates are shown in Figure 5a. Notably, the R6G characteristic peaks remained visible, even at the lowest concentration of 1.0 × 10^−16^ M. Hence, it can be concluded that the detection limit of the AgNW substrate reaches 1.0 × 10^−16^ M. Moreover, it can be noted that the intensity values increase progressively with an increase in concentration across the tested concentration range. The C-C bond stretching vibration signal of the R6G molecule is reflected significantly by the typical peak spectra of 1364 cm^−1^ [36]. 

The intensity of the characteristic spectra of 1364 cm^−1^ in the range of 1.0 × 10^−8^ M–1.0 × 10^−16^ M is shown in Figure 5b. In the 1.0 × 10^−8^ M–1.0 × 10^−16^ M range, although the peak intensity values always increase with an increasing concentration, the intensity values can be divided into two segments for linear detection, depending on the magnitude of their changes. The characteristic peak Raman intensity is linearly well-related to the logarithm of R6G concentrations in the 1.0 × 10^−8^ M–1.0 × 10^−11^ M range (as shown in Figure 5b in the cyan area), and the linear relationship is as follows: *I* = 7583.6*lgC_R6G_*-8422.5, where *I* is the SERS intensity at the 1364 cm^−1^ characteristic peak, linear correlation coefficient *R^2^*= 0.9708. Meanwhile, in the 1.0 × 10^−11^ M–1.0 × 10^−16^ M range (a grey area), the intensity shows little change and shows another linear relationship related to the logarithm of R6G concentrations. This indicates that only a few of the R6G molecules adsorbed in nanogaps between AgNWs provide a weaker Raman signal, likely due to the monolayer adsorption mode in the low concentration range [37]. This shows the relationship *I* = 127.5*lgC_R6G_*-79.5, where *I* is the SERS intensity at the 1364 cm^−1^ characteristic peak, linear correlation coefficient *R*^2^ = 0.966.

The enhancement factor (EF) significantly expresses the SERS substrate’s enhancement performance. It refers to the calculated values of the average Raman signal enhancement contributed by each molecule on the SERS substrate compared to the usual signal by each molecule on an original inactive substrate [38]. Considering the fact that the R6G molecule has a maximum electronic absorption at 528 nm, the EF is calculated as 6.12 × 10^11^ under 532 nm laser excitation with a strong resonance effect (the detailed calculation for EF is described in the Supplementary Materials, Figure S2) [30,39,40]. Compared with previous work, the substrate prepared in this paper shows excellent Raman scattering enhancement ability, as shown in Table 1. The EF without the resonance effect was calculated as 2.35 × 10^6^ using a 633 nm laser excitation. A more detailed calculation is provided in Supplementary Materials, Figure S3. 

## 4. Conclusions

In conclusion, with a simple homemade device, AgNWs with a diameter of 45 nm can be self-assembled into films at a water-oil-air three-phase interface at an appropriate evaporation rate. We achieved a SERS substrate with highly aligned arrays through the modulation of the concentration of AgNWs in ethanol and hexane dispersions. With R6G as probe molecules, the aligned AgNW arrays, functioning as excellent SERS substrates, demonstrated a good signal reproducibility with RSD as low as 4.7%. Furthermore, the DL of the substrates was as low as 10^−16^ M. It displayed an ultrahigh sensitivity close to the single molecule detection level, with an EF as high as 6.12 × 10^11^ under a strong resonance excitation. The EF without the resonance effect was calculated as being 2.35 × 10^6^ when using a 633 nm laser excitation. In addition, between the highly aligned AgNWs, abundant nanogaps were characterized as uniform and reliable hotspots by SEM and FDTD simulations. Therefore, these ordered arrays of fine nanowires on Si materials have broad application prospects as facile and robust SERS substrates.

## Figures and Tables

**Table 1:** The comparison with the results of previous studies.

SERS Substrate Structure	Detection Limit	EF	References
Ordered AgNWs	5 × 10^−6^ M R6G	6.6 × 10^5^	[41]
Ordered AgNWs	5 × 10^−9^ MR6G		[42]
Aligned AgNWs	10^−10^ M MG ^1^	4 × 10^3^	[43]
Aligned AgNWs	10^−10^ M R6G	10^4^	[30]
AuNR–AgNW nanocomposite ^2^	10^−4^ M DTTCI ^3^		[44]
Aligned AgNWs	10^−16^ M R6G	6 × 10^11^	Our work

^1^ Malachite Green—MG; ^2^ Au nanorods co-assembled with Ag nanowires; ^3^ 3,3′-Diethylthiatricarbocyanine Iodide—DTTCI.

## Data Availability

The authors confirm that the data supporting the findings of this study are available within the article [and its Supplementary Materials].

## Supplementary Materials

The following supporting information can be downloaded at: www.mdpi.com/article/10.3390/nano13081358/s1, Figure S1: SEM images of uncleaned AgNW morphology; Figure S2. (a) 1×10^−16^ M R6G SERS spectrum on the optimal AgNWs SERS substrate and bulk R6G Raman spectrum on Si substrate; (b–c) FDTD simulation of the electric field distribution of AgNWs arrays in the xy and xz cross-sections; Figure S3. The Raman spectra of solid R6G samples tested on silicon wafers and the SERS spectra of 10^−10^ M R6G samples tested on AgNWs substrates with a 633 nm laser; Figure S4. The cross section of AgNWs film prepared from 1mg/ml sample. References [30,39,40] are cited in the supplementary materials.
