# Supplementary material for: Self-Assembly of Silver Nanowire Films for Surface-Enhanced Raman Scattering Applications
Source: Nanomaterials (Basel). 2023 Apr 13;13(8):1358. doi: 10.3390/nano13081358 (PMC10146873; doi:10.3390/nano13081358)
Supplement: Supplementary file 1 [file nanomaterials-13-01358-s001.zip › nanomaterials-2295364-supplementary.pdf]

nanomaterials

Self-Assembly of Silver Nanowire films for Surface-Enhanced Raman Scattering Applications

Yanzhao Pang [1,2], and Mingliang Jin [1,2,*]

1 South China Academy of Advanced Optoelectronics, South China Normal University Guangdong 510006, China; pangyz@m.scnu.edu.cn
2 International Academy of Optoelectronics at Zhaoqing, South China Normal University Zhaoqing 526060, China; e-mail@e-mail.com
* Correspondence: jinml@m.scnu.edu.cn

Supplementary Materials:

The shape of the purchased original AgNW is shown in Figure S1.

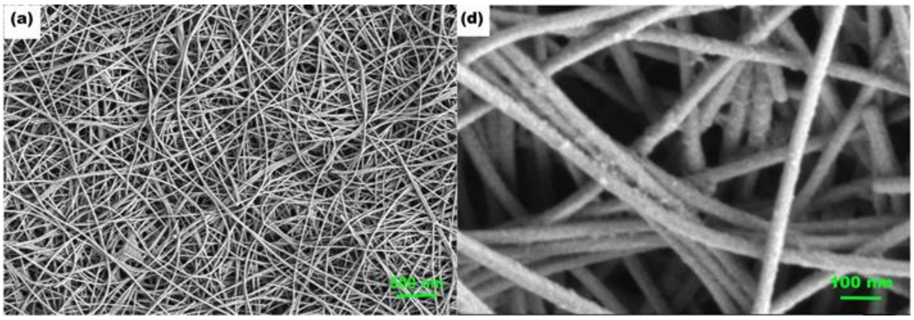

Figure S1: SEM images of uncleaned AgNW morphology

EF:
The following equation can calculate the EF of AgNWs substrate:

$$EF = (I_{SERS}/N_{SERS})/(I_{Nor}/N_{Nor}) \quad (1)$$

$$N_{nor} = \frac{\rho_{R6G} \times V_{Laser}}{M_{R6G}} \times N.A. \quad (2)$$

$$N_{SERS} = \frac{A_{SERS}}{A_{dish}} \times V_{R6G} \times C_{R6G} \times N.A. \quad (3)$$

$$V_{Laser} = S_{laser} \times H_{Laser} \quad (4)$$

Where I_{SERS} and I_{Nor} are SERS intensity of 1× 10^{-16} M R6G on AgNWs substrate and Raman in-tensity of bulk R6G on Si substrate at 1364 cm^{-1} characteristic peak, respectively. N_{SERS} and N_{Nor} represent the number of R6G molecules contributing to the SERS intensity on the AgNWs substrate and the bulk R6G substrate without enhancement in the laser-irradiated area, respectively. Figure S2. (a) shows the SERS spectrum of 10^{-16} M R6G on AgNWs substrate and the Raman spectrum of solid R6G on Si substrate. The spectra in-tensity at 1364 cm^{-1} is measured separately by deducting these baseline values. Therefore, I_{SERS} and I_{Nor} are 127 and 685, respectively. We obtained the N_{Nor} value using equation 2. ρ_{R6G} is the solid density of R6G, which is 1.1702 g cm^{-3}. M_{R6G} is the molecular mass of R6G, which is 479. V_{Laser} is the volume of laser irradiation calculated by equation 4. The S_{laser} is the laser spot size which is 10 μm diameter area. H_{Laser} is the effective focusing height of the laser beam, which is 26 μm. We focused the laser beam using a sample stage, and during the focusing process, we recorded the position of the sample stage when observing the Si Raman peak. The sample stage was then moved further until the Si peak disappeared, and the position of the sample stage was recorded at this time. The total distance moved by the sample stage was divided by 2 to obtain the height of the laser focus. The N_{Nor} value is approximately equal to 3× 10^{12}. We evaluated the N_{SERS} value by using the

area of the laser-irradiated SERS substrate, the total size of the substrate of 0.25 cm^2, the total volume of the R6G solution of 5 ml, and the total number of molecules in the 1×10^{-16} M R6G soaking solution. We assumed that all the molecules in the soaking solution were uniformly distributed on the bottom of the petri dish. The irradiation area of the laser spot is calculated to be 78.5 μm^2 with a spot diameter of ~10 μm, so the N_{SERS} at 1×10^{-16}M concentration corresponds to 0.904. Hence, EF is calculated as 6.12×10^{11}. During the calculation of N_{SERS}, we assume that all molecules are evenly adsorbed on the bottom of the petri dish. We calculate this based on the proportion of the laser spot size area to the area of the petri dish bottom. This assumption leads to our calculated N_{SERS} numbers being much larger than the actual numbers, resulting in an overall EF number smaller than the real number.

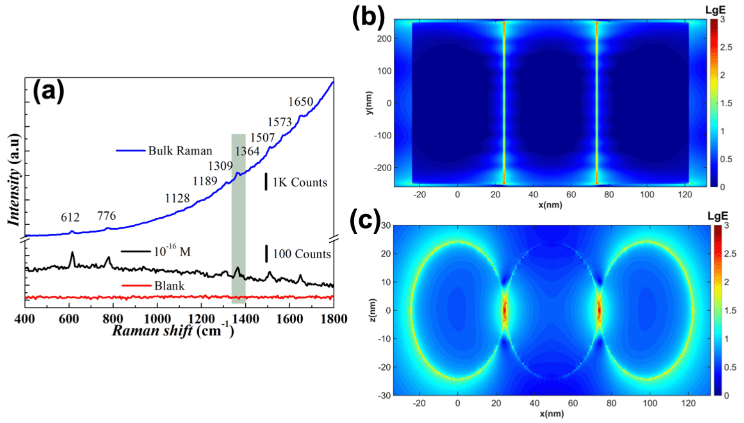

Figure S2. (a) 1×10^{-16} M R6G SERS spectrum on the optimal AgNWs SERS substrate and bulk R6G Raman spectrum on Si substrate; (b-c) FDTD simulation of the electric field distribution of AgNWs arrays in the xy and xz cross-sections.

To investigate the SERS enhancement mechanism of Ag NWs arrays, we simulated the electric field distribution at the interstices of AgNWs by the FDTD method. Figure 5(b-c) shows that the electric field enhancement mainly occurs at the gap between the nanowires. This result is consistent with previous research results[1,2]. Moreover, there is a more vigorous enhanced electric field intensity at the terminal position of the nanowires. The previous study showed the extra lightning rod effect at the endpoint of the nanowires[3]. The enormous magnitude of the enhanced electric field is about 300. Thus, the corresponding SERS enhancement factor $|E|^4$ is approximately equal to 8.1×10^9. The R6G molecule has a strong chemical enhancement ability; its range is 10^2-10^4. Therefore, the overall EF is 8.1×10^{11}-8.1×10^{13}. This is close to the result of the previous calculations**.**

Considering the R6G molecule has a maximum electronic absorption at 528 nm, the EF is calculated as 7.2×10^{11} under 532 nm laser excitation with a strong resonance effect. The EF without the resonance effect was calculated as 2.35×10^6 using the 633 nm laser excitation. A more detailed calculation is provided in support information S3.

The following sentence was added in the support information S3:

The maximum absorption spectrum peak of R6G is between 500-550 nm, so the EF calculated under 532 nm laser excitation is also called resonance EF. To calculate non-resonance absorption EF, we excited R6G using a 633 nm laser. The Raman spectra were obtained using a Renishaw inVia Raman Microscope (Ren-ishaw Co., Ltd., UK). A 633 nm laser with a power of approximately 0.12 mW was directed onto the sample through a 50× objective lens. The laser spot had a diameter of about 1.50 μm on the substrate, and each Raman spectrum was collected with an accumulation time of 0.3 s. The Raman spectra of solid R6G samples tested on silicon wafers and the SERS spectra of 10^{-10} M R6G samples tested on AgNWs substrates are de-picted in Figure S3. The EF number was calculated using equations (1)-(4). Where I_{SERS} and I_{Nor} are SERS intensity of 1×10^{-10} M R6G on AgNWs substrate and Raman in-tensity of bulk R6G on Si substrate at 1507 cm^{-1} characteristic

peak, respectively. Therefore, I_{SERS} and I_{Nor} are 35 and 940, respectively. Since the Raman microscope is changed, the number of A_{Laser} is calculated as 1.77 μm2 with 1.5 μm as the laser beam diameter. The H_{Laser} was adjusted to 17 μm. The N_{Nor} value is approximately equal to 4.42× 10^{10}. The N_{SERS} was also recalculated at 1×10^{-10} M concentration corresponding to 754. Hence, EF is calculated as 2.35X10^6. During the calculation of N_{SERS}, we assume that all molecules are evenly adsorbed on the bottom of the petri dish. We calculate this based on the proportion of the laser spot size area to the area of the petri dish bottom. This assumption leads to our calculated N_{SERS} numbers being much larger than the actual numbers, resulting in an overall EF number smaller than the real number.

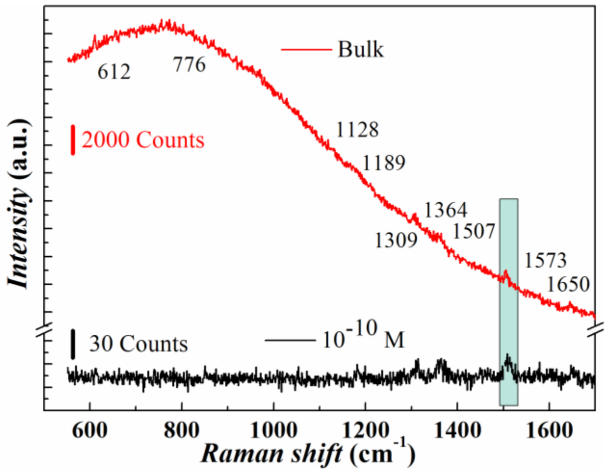

Figure S3. The Raman spectra of solid R6G samples tested on silicon wafers and the SERS spectra of 10^{-10} M R6G samples tested on AgNWs substrates with a 633 nm laser.

The average diameter of the nanowire is 45nm ±10 nm (support information S4).

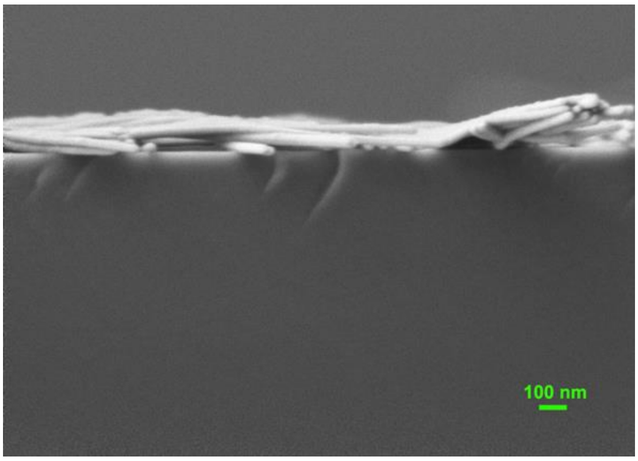

Figure S4. The cross section of AgNWs film prepared from 1mg/ml sample.

References

1. Chen, S.; Li, Q.; Tian, D.; Ke, P.; Yang, X.; Wu, Q.; Chen, J.; Hu, C.; Ji, H. Assembly of long silver nanowires into highly aligned structure to achieve uniform “Hot Spots” for Surface-enhanced Raman scattering detection. *Spectrochimica Acta Part A: Molecular and Biomolecular Spectroscopy* **2022**, *273*, 121030, doi:https://doi.org/10.1016/j.saa.2022.121030.
2. Hu, H.; Wang, Z.; Ye, Q.; He, J.; Nie, X.; He, G.; Song, C.; Shang, W.; Wu, J.; Tao, P.; et al. Substrateless Welding of Self-Assembled Silver Nanowires at Air/Water Interface. *Acs Appl Mater Inter* **2016**, *8*, 20483-20490, doi:10.1021/acsami.6b06334.
3. Du, C.; You, Y.; Chen, T.; Zhu, Y.; Hu, H.; Shi, D.; Chen, H.; Shen, Z. Individual Ag nanowire dimer for surface-enhanced Raman scattering. *Plasmonics* **2011**, *6*, 761-766.
